# Initial clubfoot treatment in Sweden from 2016 to 2019: A national register study

**DOI:** 10.1371/journal.pone.0305900

**Published:** 2024-06-26

**Authors:** Arne Johansson, Henrik Wallander, Anna-Clara Esbjörnsson

**Affiliations:** 1 Department of Orthopaedics, Skaraborg Hospital, Skövde, Sweden; 2 Departments of Orthopedic Surgery, Gävle Hospital, Gävle, Sweden; 3 Department of Clinical Sciences Lund, Orthopaedics, Skane University Hospital, Lund University, Lund, Sweden; Iran University of Medical Sciences, ISLAMIC REPUBLIC OF IRAN

## Abstract

**Background:**

This study aimed to describe the initial treatment of clubfoot deformity in Sweden using a national cohort. Secondarily we aimed to analyse the results of the initial treatment in relation to foot severity and additional diseases.

**Methods:**

A national register, the Swedish Pediatric Orthopedic Quality Register, was used to extract data on children born with clubfoot in 2016–2019. Children with a registered evaluation after initial treatment were included. Data on deformity severity (Pirani score), casting treatment, and achillotenotomy were extracted. For children with bilateral clubfeet, one foot was included in the analysis.

**Results:**

A total of 565 children were included in the analysis. Of these, 73% were boys and 47% had bilateral clubfeet. Children with isolated clubfoot required a median of six casts to correct the deformity, while children with non-isolated clubfoot needed a median of eight casts. Seventy-seven percent underwent an achillotenotomy. Residual deformities of 0.5 or above (often soft-tissue issues) according to the Pirani score were noted in 23% (isolated clubfoot) and 61% (non-isolated clubfoot) after initial treatment.

**Conclusions:**

We have described the initial clubfoot treatment of children born with isolated or non-isolated clubfoot in Sweden based on data from a national register. The initial treatment was performed to a large extent according to the Ponseti method and international recommendations. Moreover, we discuss the usefulness of the Pirani score in classifying clubfoot deformity after treatment.

## Introduction

Congenital talipes equinovarus (CVET), from now on referred to as clubfoot, is a common congenital orthopedic pediatric foot deformity [1]. Clubfoot, caused by increased collagen synthesis resulting in thick and tight ligaments and less flexible muscles, is characterised by equinus of the ankle, varus of the hindfoot, as well as cavus and forefoot adduction with associated atrophy of the calf muscles [2]. Clubfoot commonly presents as isolated congenital, even though the condition may be associated with other conditions such as myelomeningocele or arthrogryposis [3]. The cause is thought to be multifactorial, including both genetic and environmental factors [3]. In Sweden, about 1.35 children/1,000 live births are born with clubfoot, including both isolated and non-isolated cases. Of the children with isolated clubfoot, about 74% are boys and 47% have bilateral involvement [4]. Eight per cent of the children born with clubfoot in Sweden between 2016 and 2019 were reported to the national clubfoot register as having clubfoot in combination with other diseases, e.g. spina bifida or arthrogryposis, referred to in this article as non-isolated [4, 5].

The Ponseti method is currently regarded as the gold standard for the treatment of both isolated and non-isolated clubfoot [6]. Initial treatment, the theme of this article, involves weekly stretches, manipulations and casting, accompanied by achillotenotomy when needed [7]. This is followed by brace treatment for four to five years [8]. Initial treatment typically starts within the first weeks of life. An early start is encouraged, even though good results have been shown after treatments starting later in life [9, 10]. According to the Ponseti method, five to seven casts are typically needed to correct a clubfoot in a child with an isolated clubfoot [11]. For those with non-isolated clubfoot or clubfeet with atypical signs, a modified casting technique and sometimes a larger number of casts are required [12, 13]. The Ponseti protocol has improved clubfoot treatment, with a described drop in surgical interventions, above achillotenotomy, within initial treatment [12, 14, 15]. However, a large number of the children treated according to the Ponseti method will experience a relapse to some degree [16–20].

Clubfoot deformity is often classified at presentation and after casting treatment by either the Pirani or the Dimeglio score. It has been debated whether a foot, after casting treatment, must obtain a score of 0 on the Pirani scale to be considered to be fully corrected [21]. Moreover, the predictive value of both the Pirani and the Dimeglio scores on the number of casts has been evaluated with ambiguous results and often in favour of the Dimeglio score [11, 22–24].

In 2015, the Swedish Pediatric Orthopedic Quality Register (SPOQ) was designated as a national quality register and covers five common pediatric diseases of which clubfoot is one [5]. The general aim of this national prospective total cohort register is to obtain generalisable knowledge and to improve treatment and outcomes for all children born with clubfoot. In this national register, foot deformity before and after treatment and details of initial treatment, e.g. number and type of cast, are reported according to a standardised protocol.

### Aim

The primary aim was to describe the initial casting treatment for clubfoot deformity in Sweden using a national cohort. Secondarily, we aimed to analyse the results of the initial casting treatment in relation to the severity of clubfoot deformity and additional diseases.

## Materials and method

At birth, upon suspicion of clubfoot, the child is referred to one of the 28 pediatric orthopedic centres treating clubfoot. Twenty-seven of these centres register in the SPOQ and the coverage of the register since 2017 is 96%. Since practically all the children in Sweden are born in hospital, there are virtually no undiagnosed cases. To validate the number of children with clubfoot registered in the SPOQ, these numbers were compared with those in the Swedish national patient register and the average national completeness was 83.4% in 2019 [5]. Everyone living in Sweden has a personal ID number that is exclusive to the individual and does not change during a person’s life.

### Ethics statement

The study was approved by the Swedish Ethical Review Authority, D nr: 2019–04989. Details applying to this ethical approval are given elsewhere [4]. Unidentified data was accessed 11 th of June 2020, and participants could not be identified by the authors.

### Participants

After a clubfoot diagnosis, the child is enrolled in the SPOQ, administered by the treating hospital. The inclusion and exclusion criteria in the SPOQ are described in detail elsewhere [4]. Children with isolated or non-isolated clubfoot were registered. The gold standard for the treatment of children with clubfoot in Sweden between 2016 and 2019 was the Ponseti Method [25]. In this prospective cohort study, all 612 children with a total of 905 clubfeet registered in the SPOQ between 1 January 2016 and 31 December 2019 were included. Details of birth prevalence and foot involvement from each year are described elsewhere [4].

The following variables were extracted from the SPOQ: the number of children with isolated clubfoot and non-isolated clubfoot, gender, uni- or bilateral involvement, the presence of atypical signs before the start of treatment, the Pirani score at presentation and after the last cast was removed, the number and type of casts and the number of performed achillotenotomies.

The Pirani score is a disease-specific foot deformity classification system, scoring the foot deformity from 0, no foot deformity, to 6, maximum foot deformity [26–28]. Feet scoring less than 1 are classified as positional clubfeet or other minor foot deformities and are not possible to register in the SPOQ [5]. All clubfeet were classified according to the Pirani score at presentation and after the last cast was removed.

In the SPOQ, clubfeet associated with some other disease (non-isolated clubfoot) are reported upon entry in the register in one of the following categories: arthrogryposis multiplex congenital Q74.3, spina bifida Q05.9, congenital malformation syndromes predominantly involving limbs Q87.2, neurological diseases (not specified) and other (not specified). The number of clubfeet associated with other diseases were adjusted based on updated reports at the one-year follow-up.

To assess the presence of atypical signs, the SPOQ provides several illustrations to help clinicians to report this correctly. The presence of atypical clubfoot is registered upon entry, before the start of treatment, and is defined by two compulsory signs: distinct cavus and deep plantar creases crossing over to the lateral side of the foot, and five additional signs: short and stubby foot, extended greater toe, deep posterior crease, distinct equinus of > 60°, or short calf muscles < 1/3 of calf length. In this study, atypical feet were defined as isolated congenital clubfoot if no other diagnosis related to clubfoot was registered [5]. In SPOQ, the term “atypical clubfoot” is exclusive to feet that are atypical at presentation and it is not possible to report feet developing atypical or complex signs during treatment in the register.

### The casting procedure

The gold standard for clubfoot treatment in Sweden is the Ponseti method, including the Ponseti casting technique, followed by treatment with a foot abduction brace as the first choice. To the best of our knowledge, no pediatric orthopedic center treated children according to alternative methods between 2016 and 2019. In one of the 27 centers the casings were mainly performed by physiotherapists, in all others by paediatric orthopedic surgeons. In this study, initial treatment was defined as the first treatment on the untreated clubfoot including weekly manipulations of the deformities with subsequent casting and, when needed, an achillotenotomy. Hence, the total number of reported casts also includes casts applied after the achillotenotomy. An evaluation of the maintenance phase (braces) is not included in the scope of this article.

### Data and statistical analyses

Statistical analyses were performed using the Statistical Package for Social Sciences, version 25 (SPSS Inc., Chicago, IL, USA).

Demographics and disease characteristics are described using median and minimum and maximum, frequency or per cent. Differences in the Pirani score and the number of casts used between children with isolated clubfoot and non-isolated clubfoot were evaluated using the Mann-Whitney U test.

A chi^2^ test was used to estimate differences in the proportion of boys/girls, uni-/bilateral involvement and the number of feet with atypical signs between children with isolated clubfoot and those with non-isolated clubfoot.

A Spearman rank-order correlation test was used to analyse the correlation between the Pirani score at diagnosis and the number of casts.

To account for the effect of bilateral disease when evaluating the treatment, only one foot from children with bilateral disease was included. The right or left foot was included for every second child based on the inclusion number in the register, allowing an even spread across centres and years. Differences were considered statistically significant for p-values of < 0.05.

## Results

### Participants

Of the 612 children registered in the SPOQ between 2016 and 2019, a clubfoot status after the initial treatment of 565 children was reported and in 47 children registration after initial treatment was missing. Of these 47 children, 57% had bilateral involvement, 74% were boys and 14% had non-isolated clubfoot. Hence, children not reported to the SPOQ after initial treatment had a non-isolated clubfoot and bilateral involvement to a greater extent.

There were no statistically significant differences between genders for foot involvement, the proportion of atypical feet or achillotenotomies, Pirani score pre- and post-treatment, or the average number of casts, consequently, males and females will be considered one group in the statistical analyses.

Of the 565 included children, 524 had isolated clubfoot. Forty-one children had non-isolated clubfoot; arthrogryposis multiplex congenita (6 children), spina bifida (5 children), congenital malformation syndromes predominantly involving limbs (14 children), neurological diseases (6 children) and other “not specified” (10 children). The demographics of the included children are shown in Table 1.

**Table 1:** Demographic description of the included children with clubfoot.

	Total population	Isolated clubfoot	Non-isolated clubfoot	Differences between children with isolated and non-isolated clubfoot
	(n = 565)	(n = 524)	(n = 41)	
Boys (n (%))	410 (73)	388 (74)	22 (54)	**0.01** ^1^
Bilateral (n (%))	265 (47)	224 (43)	24 (59)	0.75^1^
Atypical clubfoot (n (%))	37 (7)	25 (5)	12 (30)	**<0.001** ^1^
PS at diagnosis (median (min, max)	4.5 (1, 6)	4.5 (1, 6)	5.5 (1.5, 6)	**<0.001** ^2^

n; number of clubfeet/children, PS; Pirani score.

^1^ Chi^2^ test

^2^ Mann-Whitney U test

### Age at start of treatment

Children with isolated clubfoot started treatment earlier than children with non-isolated clubfoot (median of 12 days (IQR: 6–24) and 24 days (IQR: 10–42) respectively, p = 0.001). Of the children with isolated clubfoot, 84% started initial treatment within one month after birth and the corresponding number for children with non-isolated clubfoot was 70%.

### Atypical clubfoot

Of the 565 included children, 37 were reported to have atypical signs before starting treatment (Table 1). Of these 37 children, 25 had a diagnosis of isolated clubfoot. Sixty-eight per cent had a bilateral disease and 81% had a Pirani score of 5 or above. Atypical clubfoot was mainly reported (75% of the cases) by four different centres in Sweden, together responsible for 50% of the clubfeet. Feet with atypical signs required a median of nine casts (IQR; 7–11), which was significantly higher compared with children without atypical signs (median 6 (IQR; 5–8) (p>0.001)).

### Initial treatment

A median of six casts (IQR 5–8) was required to obtain full correction of the clubfoot deformities in children with isolated clubfoot. Children with non-isolated clubfoot required a median of eight casts (IQR 6–10) (p = 0.003). Of the 565 included children, plaster of Paris was used for initial treatment in 87% (454 children), 24 of 27 reporting units used plaster of Paris as a preferential material, while three units used synthetic casts. There was a tendency for children with non-isolated clubfoot to be more frequently treated with a synthetic cast (20%) compared with children with isolated clubfoot (13%), but this difference did not reach statistical significance. There was no statistically significant difference in Pirani score after treatment or achillotenotomy rate based on cast material.

The total Pirani score before treatment showed a weak yet statistically significant correlation with the number of casts used to correct the clubfoot deformity (r = 0.26, p<0.001).

### Achillotenotomy

**Fig 1:**
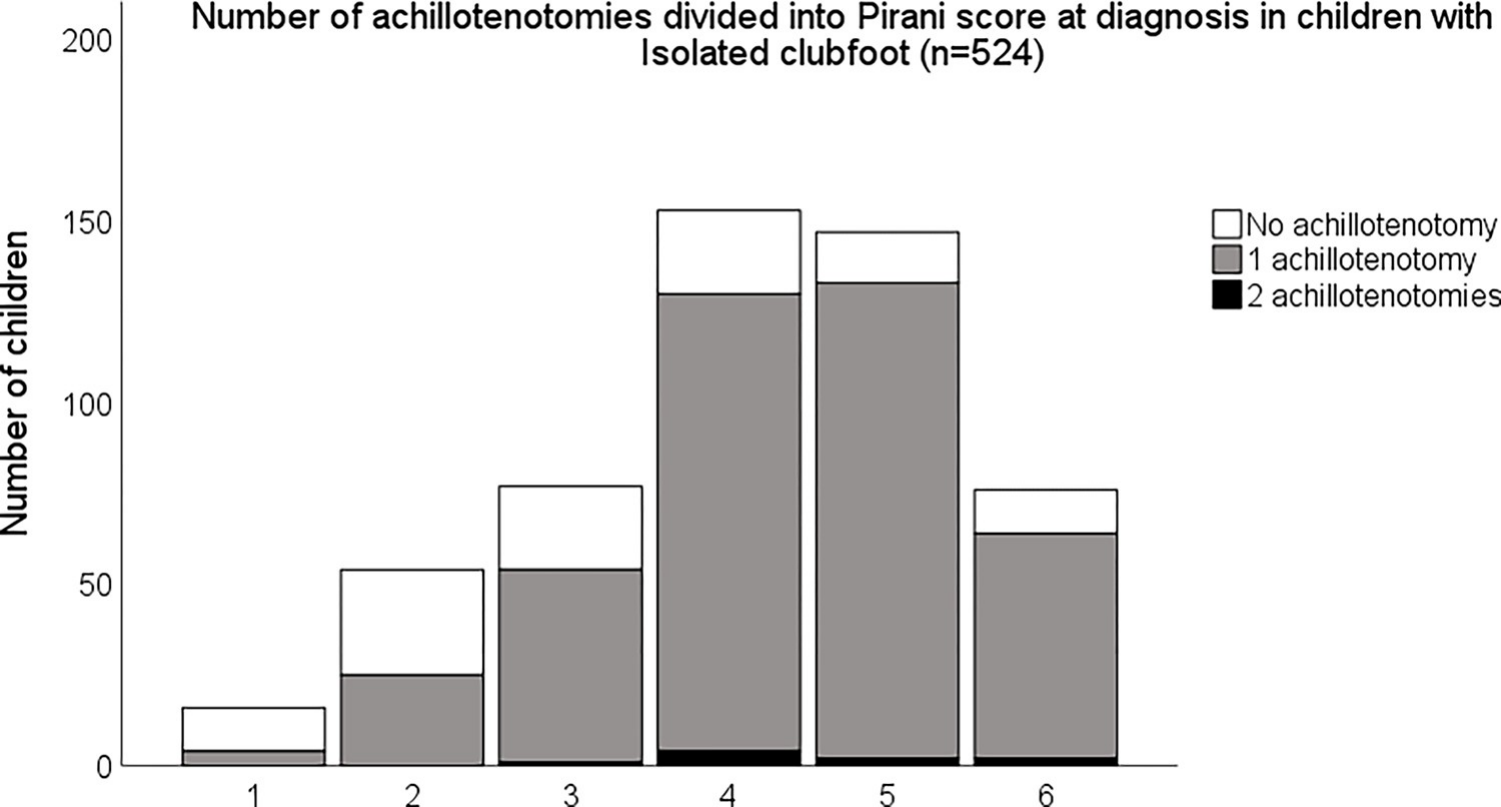
Number of achillotenotomies in children with isolated clubfoot. Children are grouped according to their Pirani score at diagnosis. The Pirani scores are grouped as follows: Pirani score 1 includes clubfeet classified as 1 or 1.5, Pirani score 2 includes clubfeet classified as 2 or 2.5 and so on. The Pirani score group of 6 includes clubfeet classified as 6.

**Fig 2:**
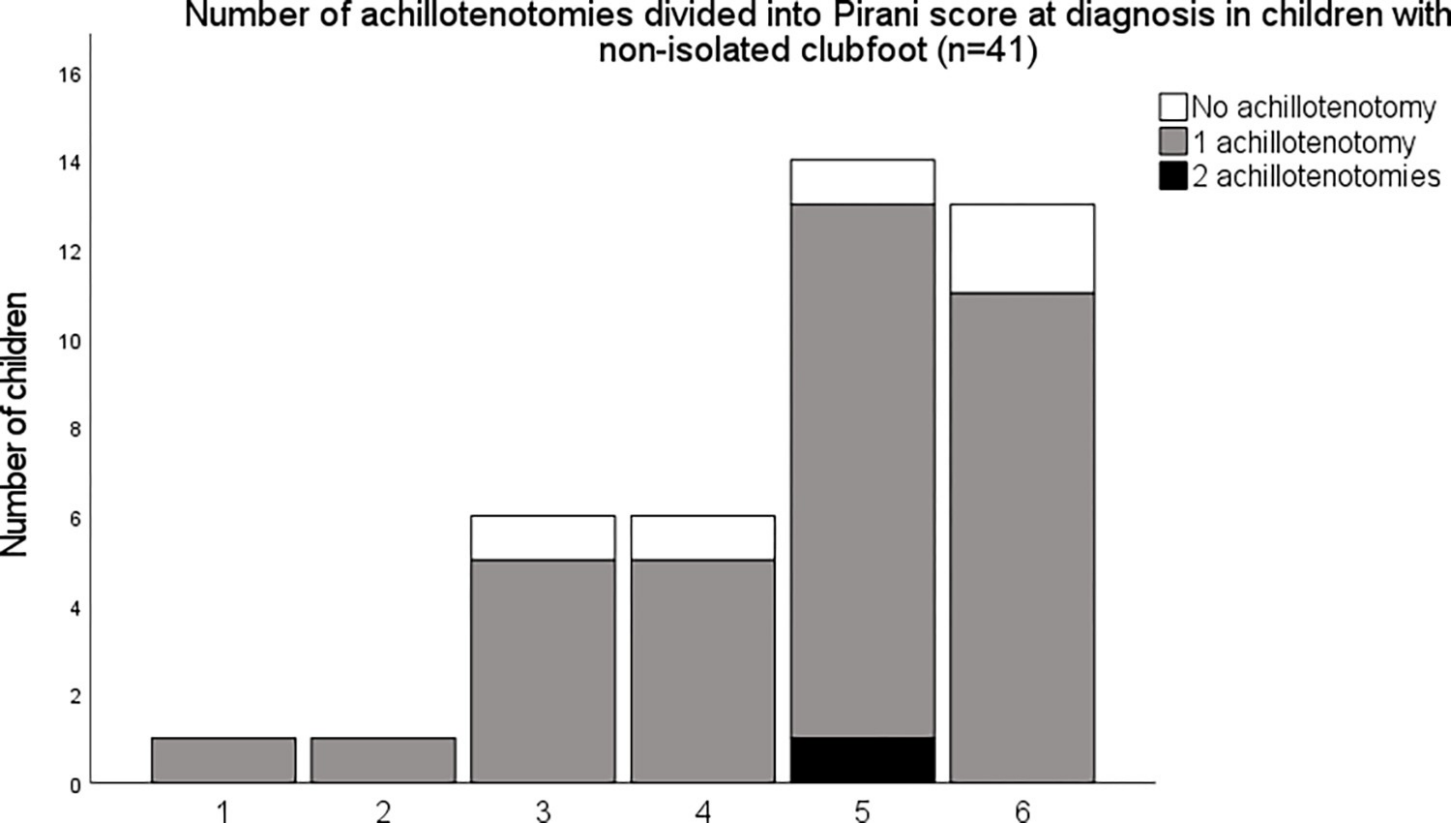
Number of achillotenotomies in children with non-isolated clubfoot. Children are grouped according to their Pirani score at diagnosis. The Pirani scores are grouped as follows: Pirani score 1 includes clubfeet classified as 1 or 1.5, Pirani score 2 includes clubfeet classified as 2 or 2.5 and so on. The Pirani score group of 6 includes clubfeet classified as 6.

Of the 565 included clubfeet, 436 (77%) underwent an achillotenotomy to correct the equinus deformity at the end of the initial treatment. Of the children with isolated clubfoot (n = 524), 76% underwent one achillotenotomy, 2% underwent two tenotomies and 22% were reported as not having a tenotomy (Fig 1). Of the children with non-isolated clubfoot (n = 41), 86% underwent one achillotenotomy, 2% (1 child) underwent two tenotomies and 12% were reported as not having a tenotomy (Fig 2). There was no statistically significant difference between the number of tenotomies between groups.

Of the 10 children requiring two achillotenotomies, only one had non-isolated clubfoot. Two were classified with atypical clubfoot at diagnosis. They all had a Pirani score of 4 or above at diagnosis. Seven of the 10 repeated tenotomies took place at either of two centres. The remaining three were spread over Sweden. The children undergoing a second tenotomy required between 12 and 21 casts (median 15) to correct the clubfoot deformities.

### The Pirani score

**Fig 3:**
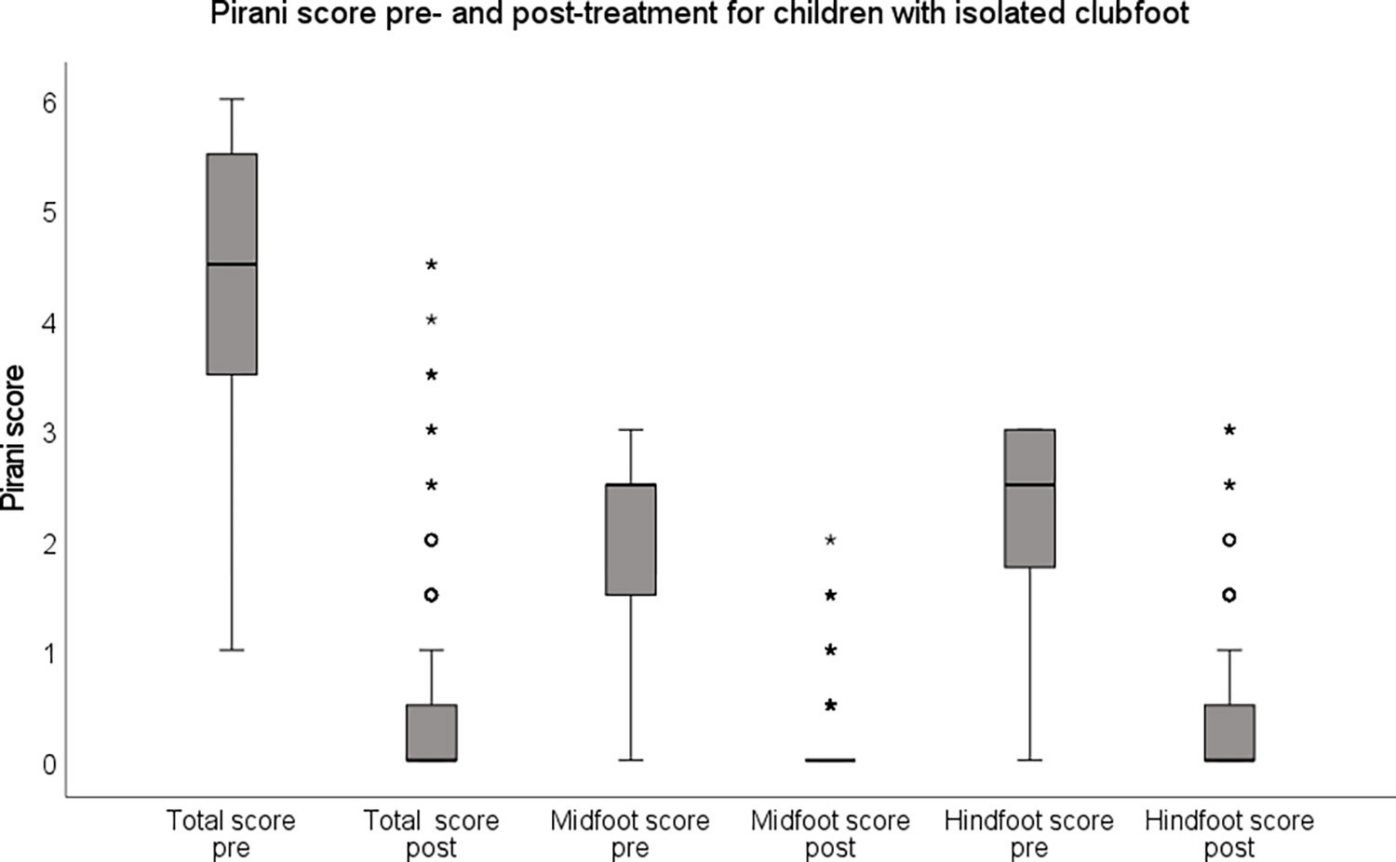
Pirani score pre- and post-treatment for children with isolated clubfoot (n = 523).

Children with isolated clubfoot had lower Pirani scores (fewer deformities) compared with children with non-isolated clubfoot both at presentation and after initial treatment (p<0.001) (Fig 3).

**Fig 4:**
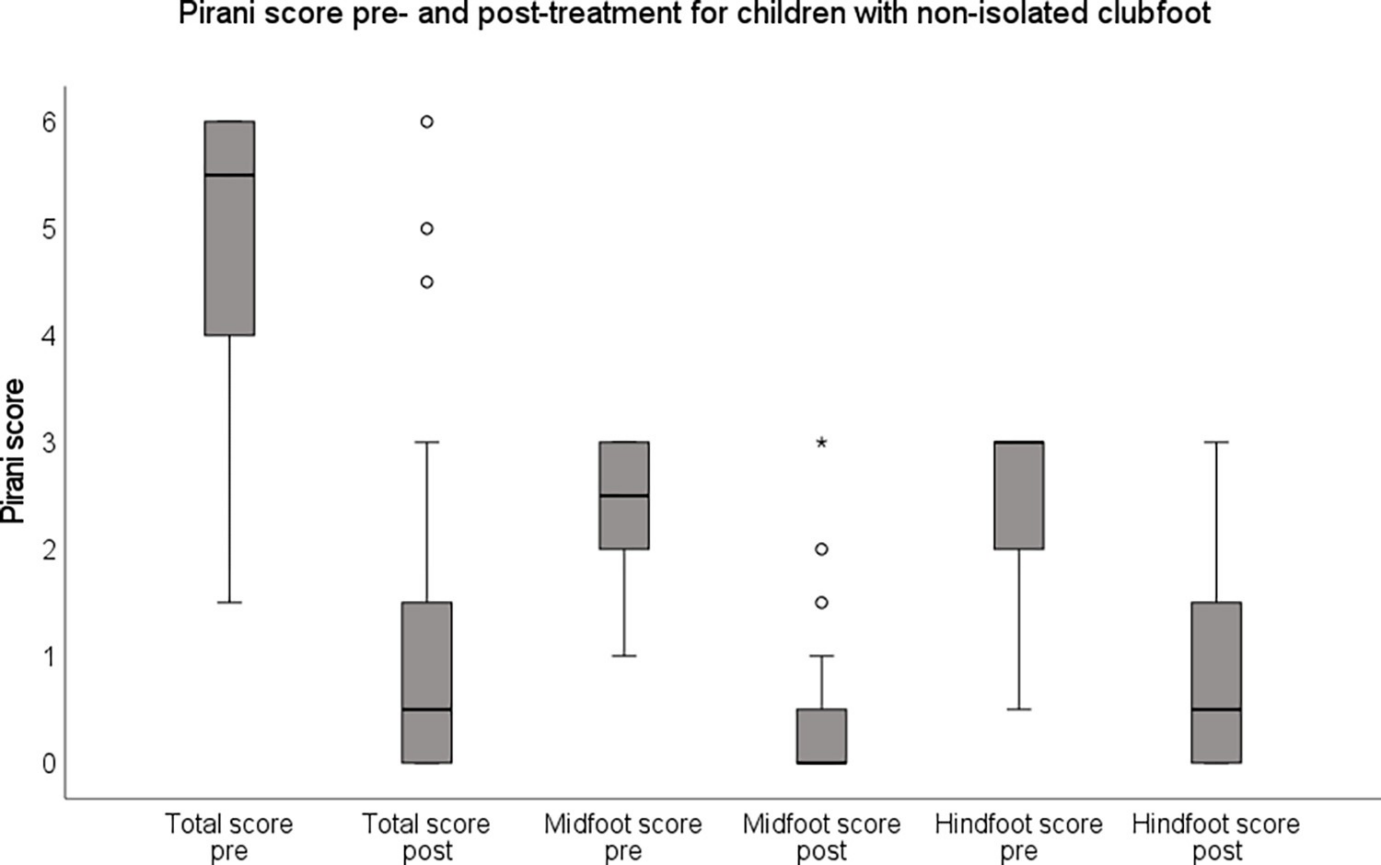
Pirani score pre- and post-treatment for children with non-isolated clubfoot (n = 41).

For children with isolated clubfoot, 349 children (67%) had a Pirani score of 0 after treatment and the highest reported Pirani score after treatment was 4.5. For the children with non-isolated clubfoot, 16 children (39%) had a Pirani score of 0 and the highest reported score was 6 (Fig 4). Residual deformities persisted to a greater extent in the hindfoot, compared with the forefoot, in both children with isolated and non-isolated clubfoot.

**Fig 5:**
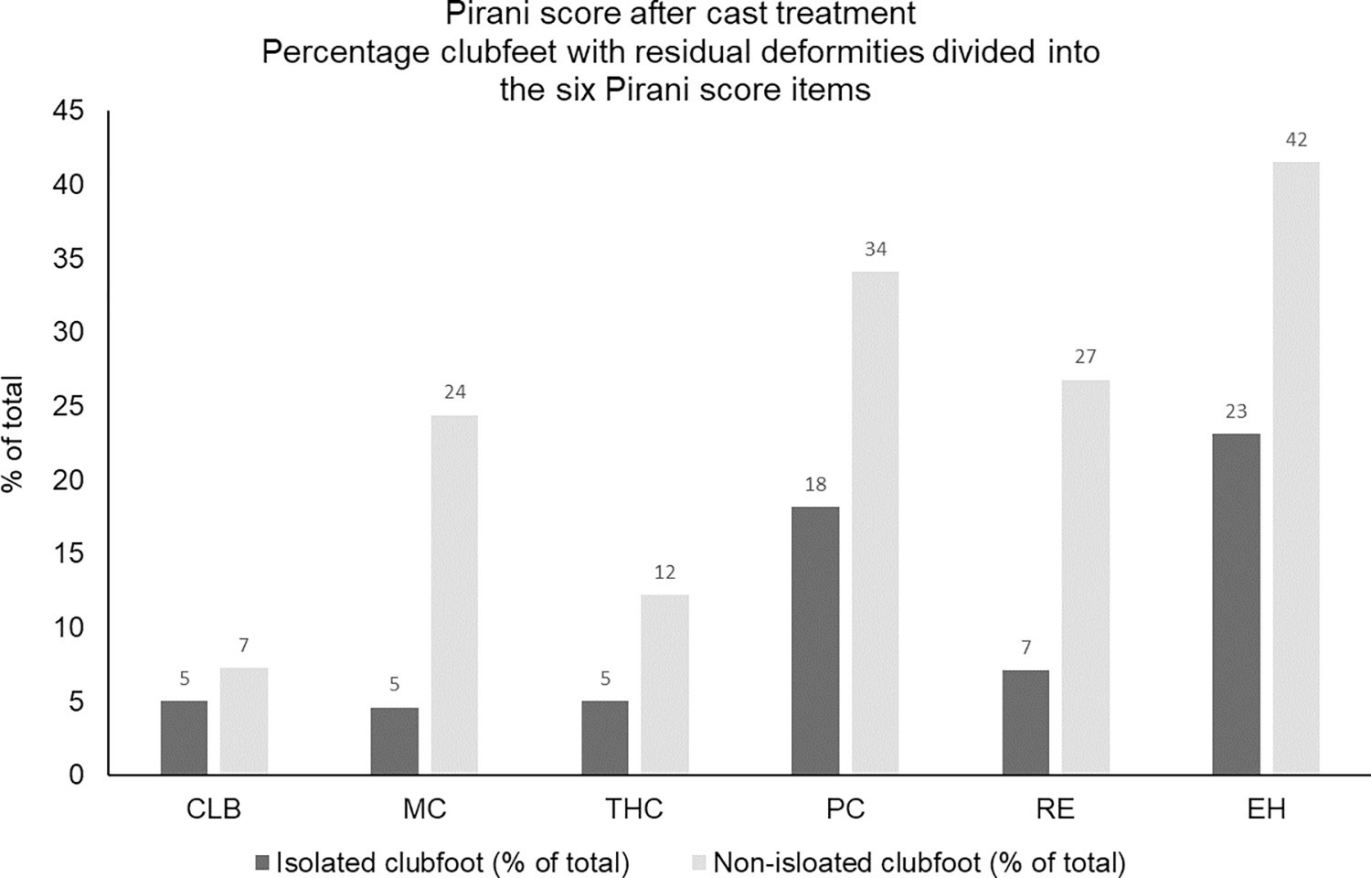
Percentage of clubfeet classified as 0.5 or 1 on each of the six items in the Pirani score after cast treatment. Divided into children with isolated (n = 523) and non-isolated clubfoot (n = 41). CLB: Curved Lateral Border; MC: Medial Crease; THC: Talar Head Coverage; PC: Posterior Crease; RE: Rigid Equines; EH: Empty Heel.

Of the six items included in the Pirani score, an empty heel and posterior crease were most often classified as not being fully corrected, followed by rigid equines (Fig 5).

## Discussion

This study, including more than 650 children with clubfeet, aimed to describe the initial clubfoot treatment in Sweden between 2016 and 2019 using a national quality register. Children with isolated clubfoot started treatment earlier and required fewer casts compared with children with non-isolated clubfoot. All feet were classified as less deformed according to the Pirani score after treatment. However, a large percentage had a post-treatment Pirani score of above 1, with a predominance of residual posterior crease and empty heel. The findings from this study define a baseline for the initial clubfoot treatment in Sweden and add to the discussion of how to evaluate the clubfoot deformity after initial treatment.

In this national cohort, a median of six casts was used to correct clubfoot deformity in children with isolated clubfoot. This is in accordance with the main body of literature and the treatment recommendations within the Ponseti method [12, 13]. Moreover, we also found that children with non-isolated clubfeet or a clubfoot with atypical signs at presentation required more casts to obtain full correction [12]. A larger number of casts have been correlated with a higher risk of relapse within the first three years of life [22]. The risk of relapse was, however, not within the scope of this article. In accordance with others, we found that children with isolated clubfoot started treatment significantly earlier compared with children with non-isolated clubfoot [9]. This finding was most probably due to birth complications, prolonged hospital stays, or additional medical evaluations for these children. No differences were found between boys and girls concerning the severity of the foot deformity or treatment regimes. In Sweden, about 74% of the children with isolated clubfoot are boys but in children with non-isolated clubfoot we note an even gender distribution [4].

Several studies have discussed, with conflicting results, the correlation between the Pirani score at presentation and the number of casts required to correct the foot deformity [22, 29]. Our study showed a weak correlation between the Pirani score at presentation and the number of casts, well in accordance with Argawal et al. (2014). Most of the feet were classified pre-treatment according to the Pirani score as 4.5–6 and required about five to seven casts, which means that the usefulness of the prediction of the number of casts can be questioned. In the parental information, it is important to emphasise that the exact number of casts and the need for achillotenotomy is difficult to predict at the start of treatment.

In this study, feet with atypical signs at presentation required more casts, independently of whether the child had an isolated or a non-isolated clubfoot. The terms “atypical clubfoot” and “complex clubfoot” are commonly used synonymously, which can make comparisons between studies challenging. In the SPOQ, the presence of atypical signs is recorded before the start of treatment in accordance with the terminology in the Ponseti International Association (PIA) Guidelines [13]. The PIA guidelines distinguish between “atypical clubfeet” and “complex clubfeet”, where the first is present at birth and the second is acquired due to complications during treatment [13, 30]. Complex signs acquired during the casting treatment are well described in the literature [31], but they are not registered in the SPOQ, which is a limitation of the register. This is also true for other adverse events (e.g. pressure areas, cast slippage and skin irritation) [32] and the number of children transferred to other hospitals due to problems during treatment. As a result, we do not know the frequency of these events. From our clinical experience, the above-mentioned events probably lead to an increased number of casts. In our cohort, most of the atypical cases were treated at four of 27 hospitals. We interpret this as atypical cases were referred to hospitals with more experience to a greater extent, but it can also be due to the under-diagnosis of atypical feet.

On average, 76% of the children with an isolated clubfoot underwent an achillotenotomy and slightly larger numbers in the non-isolated population, albeit not statistically significant, were seen. These numbers are slightly low compared with the current literature, commonly above 80% [9, 32] However, others state that over 90% of the feet require an achillotenotomy and, in the light of this, the numbers from the Swedish cohort require further investigation [13, 17]. A few children, classified as Pirani 5 or 6 in both the isolated and non-isolated groups, were reported as not having an achillotenotomy. We know from the open data from the SPOQ register that at least 5% of the children experience a postero-medial release or a posterior release after initial treatment due to residual deformity [5]. Additional surgeries, after the initial treatment including casting and achillotenotomy, are reported to the SPOQ register separately and separately after initial treatment and were not included in the data extracted for this article but would be of interest in future investigations.

What is a corrected clubfoot? According to the Ponseti directives, a corrected clubfoot has 15–20 degrees of dorsiflexion and about 60 degrees of subtalar abduction and fits well in a foot-abduction brace [7]. In addition, the cavus and forefoot adduction deformities should be corrected [7, 12]. Correction, however, is often evaluated using the Pirani score or the Dimeglio score. An empty heel and posterior crease are the items most frequently classified as still being present after full correction is achieved and the foot fits well into the brace [21]. In our study, about 20% of the isolated clubfeet and 30–40% of the non-isolated clubfeet had a residual posterior crease and/or empty heel after treatment, while the midfoot scores were much lower. A decrease in the Pirani hindfoot score is often first achieved after the achillotenotomy while the midfoot score is often normalised within the first casts [33]. The frequent presence of residual posterior crease, especially in children with non-isolated clubfoot, requires further investigations including comparison with other measures of treatment outcome such as dorsiflexion and subtalar abduction. This result is in accordance with others who have discussed the empty heel after cast treatment and the importance of basing clinical decisions relating to achillotenotomy on equinus and not on the size of the heel pad [33, 34]. Moreover, the heel pad undergoes some natural remodelling with age and weight-bearing and this should be communicated to families [33].

The gold standard for clubfoot treatment in Sweden is the standard Ponseti method and, to the best of our knowledge, all the centres in Sweden apply these principles. However, we have no control over the education or experience level of the orthopedic surgeons or physiotherapists treating the children [35] nor of whether some centres apply the accelerated Ponseti method [36]. To the best of our knowledge, achillotenotomies are most often performed under local anaesthesia, but this information is not available in the clubfoot register [5, 32]. One strength of the current study is the national prospective approach and, close to the total cohort, of the clubfoot register with well-predefined prerequisites for registration, excluding other subtypes of foot abnormality or postural clubfoot. Associated diseases as well as signs of atypical clubfeet are reported on first registration, thereby increasing the likelihood of caregivers addressing these aspects.

## Conclusions

We have described the initial clubfoot treatment for children born with isolated or non-isolated clubfoot in Sweden based on data from a national register and initial clubfoot treatment is performed to a great extent according to the Ponseti method and international recommendations. Moreover, we discuss the usefulness of the Pirani score for classifying clubfoot deformity after treatment. These numbers could serve as a baseline when planning clubfoot treatment and when evaluating the time trends for children with clubfoot.
